# Predicting postoperative pain following root canal treatment by using artificial neural network evaluation

**DOI:** 10.1038/s41598-021-96777-8

**Published:** 2021-08-26

**Authors:** Xin Gao, Xing Xin, Zhi Li, Wei Zhang

**Affiliations:** 1 The State Key Laboratory Breeding Base of Basic Science of Stomatology (Hubei-MOST) and the Key Laboratory of Oral Biomedicine Ministry of Education, School and Hospital of Stomatology, Wuhan University, 237 Luoyu Road, Wuhan, 430079 China; 2 Department of Oral and Maxillofacial Surgery, School and Hospital of Stomatology, Wuhan University, Wuhan, China; 3 Department of Endodontic, School and Hospital of Stomatology, Wuhan University, Wuhan, China

**Keywords:** Computational biology and bioinformatics, Medical research, Risk factors

## Abstract

This study aimed to evaluate the accuracy of back propagation (BP) artificial neural network model for predicting postoperative pain following root canal treatment (RCT). The BP neural network model was developed using MATLAB 7.0 neural network toolbox, and the functional projective relationship was established between the 13 parameters (including the personal, inflammatory reaction, operative procedure factors) and postoperative pain of the patient after RCT. This neural network model was trained and tested based on data from 300 patients who underwent RCT. Among these cases, 210, 45 and 45 were allocated as the training, data validation and test samples, respectively, to assess the accuracy of prediction. In this present study, the accuracy of this BP neural network model was 95.60% for the prediction of postoperative pain following RCT. To conclude, the BP network model could be used to predict postoperative pain following RCT and showed clinical feasibility and application value.

## Introduction

Root canal treatment (RCT) is the most common therapeutic method for pulp and periapical diseases. After RCT, approximately 1–58% discomforts occur, with postoperative pain as the most common^1^. The occurrence rate of moderate to severe pain after RCT is approximately 15–25%^2,3^. As the greatest concern of patients after treatment, this pain may be related to a bio-psycho-social mechanism^4^. Various factors that influence postoperative pain have been studied, including treatment methods, such as the number of sessions to complete the treatment and so on; medication used during RCT; different types of treatment, whether it is initial treatment or retreatment; host factors, such as gender, age and oral hygiene condition; degree of tissue damage; and severity and intensity of inflammatory reaction^5,6^. The frequency and duration of pain after RCT differ in various studies.

Methods for the control or reduction of pain have been successfully developed over the past few years^7,8^. However, practitioners often assess pain after RCT on the basis of personal clinical experience with no objective methods. The inaccurate anticipation of pain may have negatives effects on the planning of subsequent therapeutic schedule, thus bringing unpleasant medical experience to patients^9^. With reasonable methods to predict postoperative pain, dentists could try to alleviate the pain through controllable factors, such as changing operative details and adjusting the medication.

In terms of doctor-patient communication, a clear and visual result supported by scientific evidence seems to be more convincing to trust. Therefore, the model is also convenient for dentists to educate patients about causes of pain and help them establish correct psychological expectations for the treatment effect^10,11^. With higher negotiation efficiency, unnecessary conflicts between doctors and patients could be avoided. Therefore, further research may aim to understand the incidence of pain after RCT better.

Artificial neural network (ANN) is the most recent and rapid development in the field of nature-inspired algorithms^12^. ANN is a system based on the human brain structure and function imitation that can be applied to analyse the relationship between various predictors and their unclear relationship^13,14^. Medical results can be predicted by selecting proper neural network structures and training weight. This network has been largely developed in the field of medicine and widely used in disease diagnosis, prognosis and clinical decision^15–18^. It is reported that ANN may enable to identify the important variables and predict post-treatment pain with high accuracy. For example, researchers have proved that the model has a potential application in predicting non-ST-elevation myocardial infarction (NSTEMI) and unstable chest pain. Besides, a study used mobile health apps and machine learning models to predict pain during treatment of acute pain in sickle cell disease^19,20^. They provide us technical possibilities for predicting pain, as well as understanding of the individual physiological mechanisms of pain and treatment. Nevertheless, there are few articles about the prediction in oral medicine. The application of ANN to anticipate postoperative pain following RCT has never been reported before. In the present study, we utilized the ANN model of error BP algorithm to predict the occurrence and degree of spontaneous postoperative pain after RCT. We wish that in RCT treatment, this model could effectively improve patients' trust in dentists and help dentists make suitable decisions.

## Materials and methods

### Parameter setting and data standardisation

The functional relationship between the physical characteristics of patients, features of involved teeth, operative factors and pain after RCT was initially established. The input data of the neural network model were the parameters related to postoperative pain. The standardisation (normalisation processing) of the following 13 parameter data is illustrated in Table 1, the normalized data is between 0 and 1: patients’ gender, age and oral hygiene status as personal characteristic parameters; sites, types, degree of spontaneous pain, degree of percussion pain, periodontitis, condition of apical inflammation and pulp vitality as the characteristic parameters of the affected teeth; factors, such as root canal underfilling, root canal overfilling and root canal missing, as the operative factors during RCT.

**Table 1:** Normalisation of parameter data.

Parameter	Normalisation processing
X1: gender	Male: 0; female: 1
X2: age	≤ 20: 0; 20–30: 0.25; 30–40: 0.5; 40–60: 0.75; ≥ 60: 1
X3: oral hygiene	Good: 0; medium: 0.5; bad: 1
X4: location of involved teeth	Maxillary: 0; mandibular: 1
X5: type of involved teeth	Incisors and canines: 0; premolars: 0.5; Molars: 1
X6: degree of spontaneous pain (initial diagnosis)	No pain: 0; slight pain: 0.2; mild pain: 0.4; moderate pain: 0.6; severe pain: 0.8; extreme pain: 1
X7: degree of percussion pain of involved teeth (initial diagnosis)	No percussion pain: 0; percussionn pain ( ±): 0.25: percussion pain (+): 0.5; percussion pain (++): 0.75; percussion pain (+++): 1
X8: tooth mobility (initial diagnosis)	Not loose: 0; I-II degree loose: 0.5; II-III degrees loose: 1
X9: radiographic appearance of apex (initial diagnosis)	No shadow: 0; small shadow: 0.5; shadow at 1/2 root length: 1
X10: vitality of dental pulp (initial diagnosis) X11: root canal underfilling	Partial vitality: 0; no vitality: 0.5; corruption and necrosis of dental pulp: 1 Suitable filling ≤ 2 mm: 0; underfilling 2–3 mm: 0.5; underfilling ≥ 3 mm: 1
X12: root canal overfilling	None: 0; overfilling ≤ 2 mm: 0.5; overfilling > 2 mm: 1
13: root canal missing	None: 0; missing 1: 0.5; missing ≥ 2: 1

The classification grade of all parameters strictly follows the accepted standard. As for oral hygiene, we chose oral hygiene index-simplified (OHI-S) to measure oral hygiene condition. It contains debris index-simplified (DI-S) and calculus index-simplified (CI-S), which was proposed by Greene and Vermillion in1960 and revised in 1964^21^. We calculated the average score of DI-S and CI-S, and stipulated 0–0.5 as good, 1–2 as medium, and 2.5–3 as bad. Regarding the score of tooth mobility (TM), we have adopted a widely used clinical judgment method. I-degree TM means that tooth loose exceeds physiological mobility, but the amplitude is within 1 mm. II-degree TM means that the amplitude is 1–2 mm and III-degree TM over 2 mm. We set not loose as 0, 1–2 degree loose as 0.5, and 2–3 degrees loose as 1.

The diagnosis of percussion pain depends on patients’ subjective feelings when knocking the affected teeth^22^. According to the comparison of the reaction between the affected teeth and the normal teeth, percussion pain can be divided into five grades: percussion pain (–) means the reaction of knocking with proper force is the same as that of normal teeth; percussion pain (±) means discomfort or strange feeling is caused by percussion with appropriate strength; percussion pain (+) represents light pain is caused by heavy knocking; percussion pain (+++) means severe pain is caused by tapping; percussion pain (++) is between (+) and (+++).

Visual analogue scale (VAS) was employed for pain assessment in this experiment^23^. The pain scale was divided into five equal parts, one end corresponding to ‘no pain’, and the other end as ‘extreme pain’. Normalisation was as follows: no pain (the patients feels good, score: 0), slight pain (the patient is distracted but not feeling pain, score: 0.2), mild pain (the patient feels pain even when concentrated on work, score: 0.4), moderate pain (the patient is very upset but can still continue daily activities, score: 0.6), severe pain (the patient is forced to suspend normal activities, score: 0.8) and extreme pain (bed rest is needed instead of any kind of activities, score: 1). VAS scoring was completed by patients according to their subjective experience, under the guidance of examiners. Before patients made their choice, examiners carefully enquired about their feelings and explained the meaning of different pain ratings as detailed as possible, in order to reduce subjective errors. VAS score one week after RCT was set as the data of postoperative pain.

### Establishment of BP neural networks model

**Figure 1:**
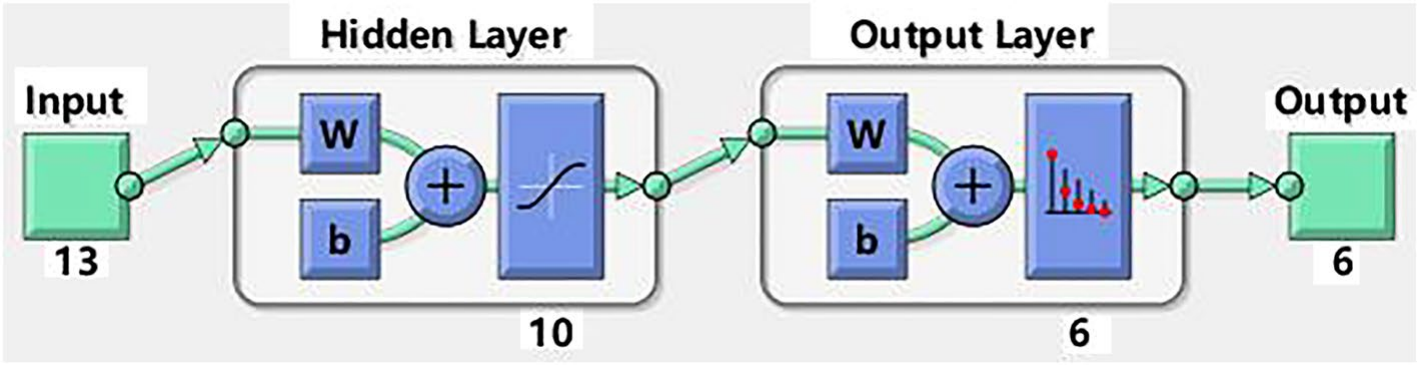
Structure of BP neural network model.

The structure of the BP neural networks is shown in Fig. 1. The number of node of input layer was 13, representing the physical condition of the patient and operative factors during RCT. And the number of hidden layer node was 10. The number of output layer node was 6, which represented the different levels of the postoperative pain of the patient. At the same time, the model improved its generalisation ability by a set of validation data samples and thus prevented overfitting the training samples.

### Sample selection and model test

The study protocol was approved by the Ethics Committee at Hospital of Stomatology, Wuhan University. All patients were informed of the clinical study and provided signed informed consent. The study was conducted in full accordance with ethical principles. A total of 300 adult patients with 300 root filled teeth who had received RCT from January 2015 to December 2018 in the Department of Endodontics in our hospital were randomly selected as subjects and provided with serial numbers.

The inclusion criteria were as follows: the affected teeth were receiving their first RCT, no contraindications for RCT was found, no psychoactive or analgesic drugs had been orally taken or infused for the past 1 month, no diabetes or immune dysfunction systemic diseases, no mental illness, female patients were not pregnant or lactating and no allergic physical constitutions. RCTs for all patients were performed by the same endodontist thrice, including pulp opening, root canal preparation and root canal filling, each at 1 week interval. Patients strictly followed the postoperative instructions. In addition, patients did not take any anti-inflammatory drugs before, during RCT and within 1 week. Experimenters evaluated the patients before, after and 1 week after RCT.

According to Table 1, the parameter data of 300 cases were normalised and counted as the input of the ANN model. Relevant data on the occurrence and degree of spontaneous pain after RCT were normalised as the output. Among them, 210 cases (patients No. 1 to No. 210) were assigned as data training samples, and 45 cases (patients No. 211 to No. 255) were used as data validation samples to improve the generalisation ability of the BP neural network model and to avoid overfitting. Furthermore, another 45 cases (patients No. 256 to No. 300) were employed as test samples to detect the predictive accuracy of this model.

## Results

### Judging the optimal performance of validation samples

**Figure 2:**
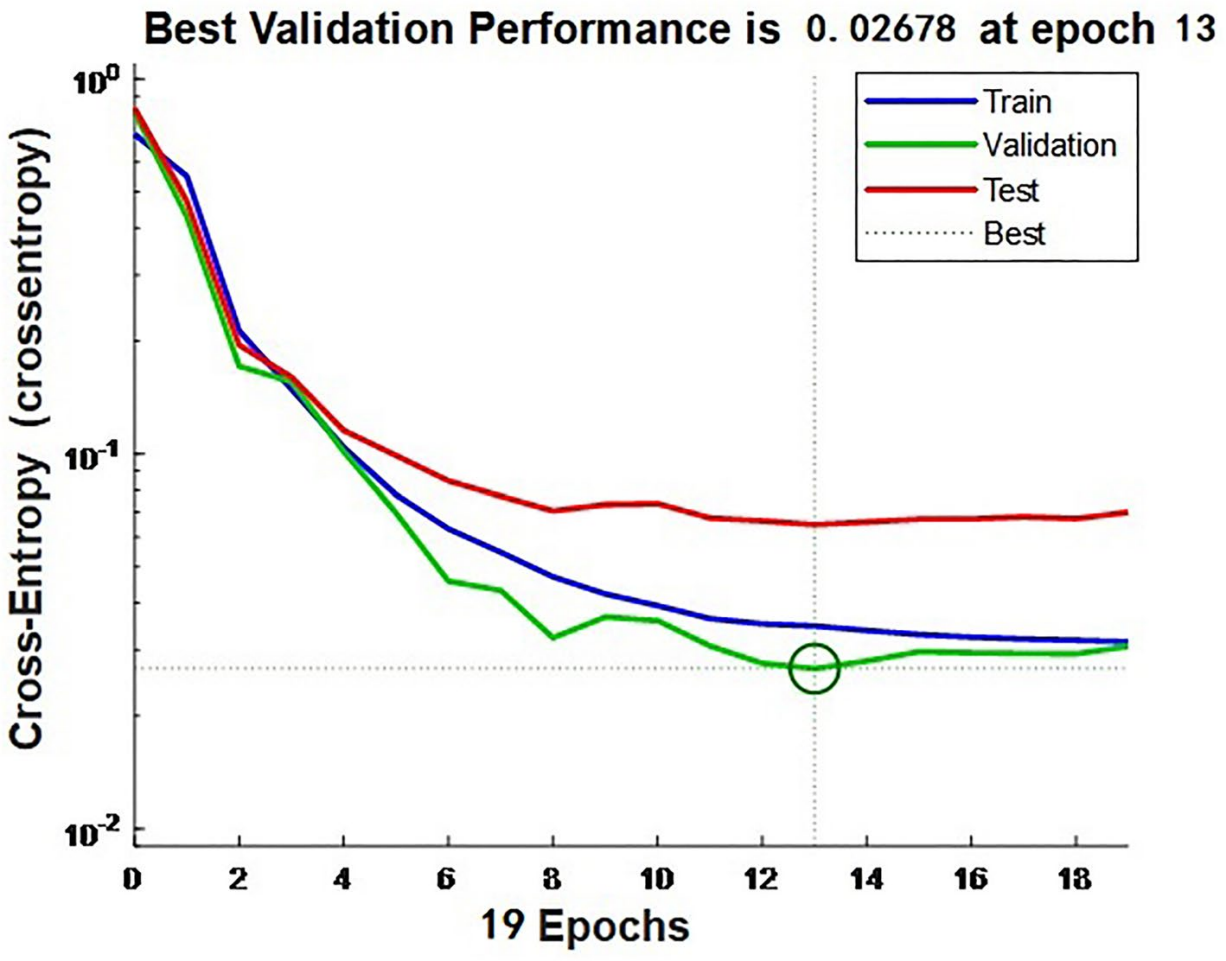
Graph of performance loss function vs. number of iterations.

The relationship curve between the performance loss function of model and iteration times is shown in Fig. 2. The blue line represents the cross-entropy over the course of training, the green line represents the cross-entropy over the course of validation, and the red line represents the cross-entropy over the course of test. The BP neural network model converged continuously in the process of 19 iterations, and the performance loss function of fitting error to test the training and validation samples decreased continuously. The optimal performance of validation samples was 0.02678, at the 13th iteration.

### Results of prediction test

**Figure 3:**
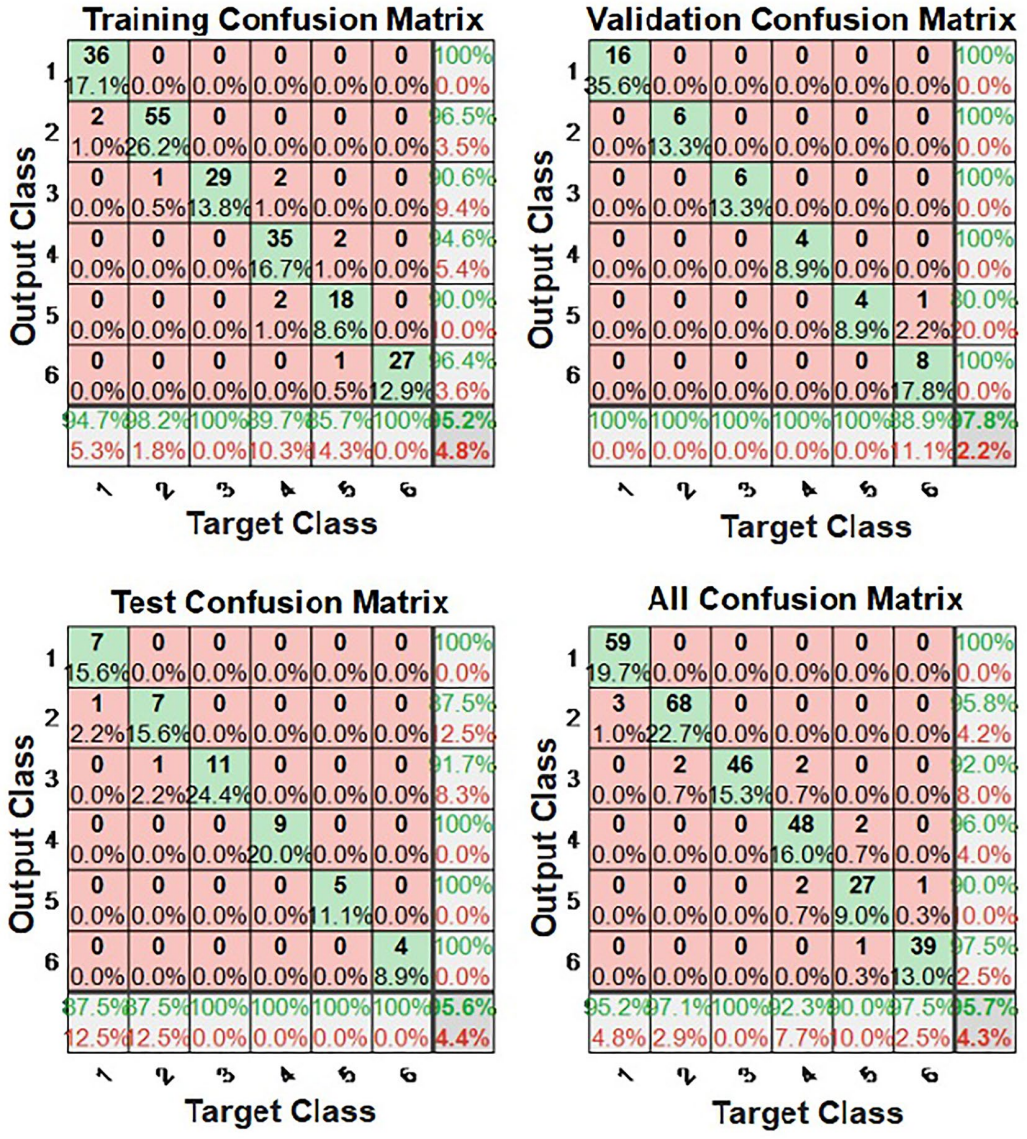
The confusion matrix of model sample.

The confusion matrix of BP neural network model for testing the training and validation samples, as well as the total data, is indicated in Fig. 3. The confusion matrix is used to evaluate the quality of the output of a classifier on the data set. The diagonal elements represent the number of points for which the predicted label is equal to the true label, while off-diagonal elements are those that are mislabeled by the classifier. The higher the diagonal values of the confusion matrix the better, indicating many correct predictions. The training accuracy of training samples was 95.2%, and the validation accuracy of the validation samples was 97.8%. In addition, the accuracy of the prediction model of test samples was 95.60%. Therefore, we supposed that the BP neural network model was able to anticipate postoperative pain of RCT.

## Discussion

With the development of endodontic treatment, the incidence of pain and swelling during RCT is only approximately 10%^24^. Patients frequently suffer from postoperative pain. Prospective clinical studies revealed that approximately 21%, 15% and 7% of patients have mild, moderate and severe pain, respectively, after RCT^25^. In clinical practice, we find that patients often evaluate their treatment effects based on subjective feelings. Due to insufficient relevant medical knowledge, it is difficult for patients to assess the treatment effect from professional aspects. With painful experiences, they tend to think that they are suffering from low-quality treatment, causing dissatisfaction and misunderstanding. However, the majority of practitioners always emphasize the control of postoperative pain instead of objective methods for accurate prediction^26–28^.

The rapid and accurate prediction of postoperative pain is necessary in root canal therapy, which can be conductive to the formulation of follow-up diagnosis and treatment plans, the adoption of preventive measures for possible pain and timely notification to patients. In this study, we used BP ANN model to estimate the postoperative pain of RCT, and the accuracy reached 95.60%, which has significant clinical value. BP-ANN is a type of classical artificial neural network, and its innovation is inspired by the structure of brain neural system. It employs machine learning based on BP algorithm, which is the basis of many complex computations. BP-ANN can be used in the medical field, including predicting body biochemical indexes and diagnosing diseases, forecasting and quantifying the synergistic effect of drugs and prognosing the results of treatment^29–32^.

For a specific case, dentists can only estimate pain after RCT based on personal clinical experiences. Multiple predictors play a role in postoperative pain. Therefore, determining how many and to what extent the factors are contributing to the occurrence of the pain is difficult. A complex non-linear relationship exists. Predicting the possibility of postoperative pain with clinical experience alone is unreliable. Thus, we must have a calculation tool that can scientifically analyse the non-linear relationship. The main advantage of ANN is that it can analyse numerous predictors (or variables) without statistical modelling and can effectively deal with non-linear problems. In addition, an essential feature of ANN is that it could learn from input data. Through the repeated training of various data, obtaining the ability to assess the results of new input data may be successful^33–35^.

The construction of neural network model must be solved first to accurately predict postoperative pain. The BP neural network contains the input, hidden and output layers. Experimenters input the variables into the input layer and obtain the desired prediction results from the output layer. Nodes in the hidden layers can analyse the nonlinear relationship between the data. Therefore, numerous training samples are first employed to establish the correct model of non-linear relationship. The core idea of the multilayer feedforward neural network of BP algorithm is to calculate the training error through forward neural network and then utilize the training errors to act on hidden-layer neurons reversely. This method adjusts the connection weight and the threshold of each neuron and continuously update to reach the minimum of training errors. BP neural network has the ability of self-study, which actually achieves a mapping function from input to output. Furthermore, mathematical theories have proven that the network contains the function of achieving any complex non-linear mappings, which makes it especially suitable for solving problems with complicated mechanisms^36–38^.

Secondly, regarding the selection of parameters and standardisation of data involved in predicting pain after RCT, this model is absolutely professional and credible. Predictors, such as gender, age, allergy history and retreatment, are closely related to the postoperative pain of RCT^39^. With age, the probability of moderate or severe pain after RCT increases^40^. Significant gender differences exist in clinical and experimental pain responses between male and female^41^, and the risk of postoperative pain in women is much higher. For the personal characteristics of patient, genetic background is also closely related^42^. However, utilizing complicated genetic types as parameters to predict pain following RCT still remains technically difficult, thus it was not used in this study. At the same time, the location, type, number of root canals, pulp vitality, preoperative pain, destruction of apical bone, acute abscess, occlusal high point and other factors of the affected teeth are considered to be involved with postoperative pain^43–45^. Clinical statistics revealed that pain after the RCT of non-molars is significantly lower than that of molars, and pain after RCT is higher in mandibular teeth than in maxillary teeth^33^ In addition, root canal underfilling, overfilling and omission, instrument selection and other operational factors are also important predictors in the postoperative pain of RCT^46–48^.

Psychological factors of postoperative pain may include a wide range of contents. For example, depression, anxiety, catastrophizing, somatization, cognitive function, and so on^49^. There are also some studies that do not mention the effect of psychological factors on pain after RCT^50^. Due to the relatively small number of studies explaining psychological mechanisms, the credibility of the analysis is reduced. Therefore, we did not include psychological factors in this study. Other studies using artificial neural network to analyze the specific influence of various psychological factors on postoperative pain could also be considered in the future.

In this experiment, we selected 13 possible variables in the personal characteristics, dental characteristics and operative factors of the patient as input parameters in the ANN model. The effect weight between these variables and postoperative pain of RCT remains unclear, but after the repeated training of numerous samples, BP neural network has automatically screened the variables internally, distributing small weight to variables that slightly contribute and large weight to variables that contributes. When the training sample is large, the influence of variable selection, parameter selection and data standardisation on the final output is minimal.

As a note, other factors that may affect postoperative pain but are not included in 13 parameters, such as different treatment methods, medication used in root canals, are all under the same control. Besides, in order to reduce inter-examiner bias and intra-examiner bias, we have trained the examiners carefully before the experiment to form an unified understanding of the diagnostic criteria. We also carried out the standard consistency test in advance to confirm that they have mastered the consistent standard. There are also some inevitable deviations. For example, traditional diagnosis of percussion pain and tooth mobility rely partially on the visual inspection and subjective reports. We are considering more accurate test methods in future study, such as 3-shape oral scanner or other intraoral measurement devices to get teeth mobility data^51,52^.

Therefore, ANN based on BP algorithm exhibits high prediction accuracy and may benefit dentists and patients in future root canal therapy. After further optimizing the measurement method, the precision of ANN model will continue to improve.

Besides, a meaningful issue worth discussion is, when the model predicts no pain but the patient reports pain, how should dentists deal with the problem? We consider that dentists could not ignore the subjective feelings of patients. Dentists need to carefully analyze the causes of pain, and combine artificial neural network prediction with personalized medical service. Artificial neural network models are our tools, not our manipulators.

Our research does have some limitations. For example, we only studied short-term pain which was one week after treatment. In future studies, longer follow-up can be considered to predict the possibility of long-term pain.

## Conclusion

Nowadays, ‘comfortable medicine’ and ‘precise medicine’ have been developed and are urgently needed by doctors. This study utilized ANN model to predict pain after RCT, provid certain clinical significance for practitioners that aim to improve the quality of RCT, establish optimized treatment plans and reduce the occurrence of medical disputes. Therefore, the proposed method might be used for clinical reference in the future.

## References

[1] Sipaviciute E, Maneliene R (2014). Pain and flare-up after endodontic treatment procedures. Stomatologija.

[2] Topcuoglu HS, Topcuoglu G, Arslan H (2018). The effect of apical positive and negative pressure irrigation methods on postoperative pain in mandibular molar teeth with symptomatic irreversible pulpitis: A randomized clinical trial. J. Endod..

[3] Lopes LPB (2019). Effect of photobiomodulation therapy on postoperative pain after endodontic treatment: A randomized, controlled, clinical study. Clin. Oral Investig..

[4] Nixdorf DR (2016). Frequency, impact, and predictors of persistent pain after root canal treatment: A national dental PBRN study. Pain.

[5] Alves Vde O (2010). Endodontic flare-ups: A prospective study. Oral Surg. Oral Med. Oral Pathol. Oral Radiol. Endod..

[6] Erdem Hepsenoglu Y, Eyuboglu TF, Ozcan M (2018). Postoperative pain intensity after single- versus two-visit nonsurgical endodontic retreatment: A randomized clinical trial. J. Endod..

[7] Suneelkumar C, Subha A, Gogala D (2018). Effect of preoperative corticosteroids in patients with symptomatic pulpitis on postoperative pain after single-visit root canal treatment: A systematic review and meta-analysis. J. Endod..

[8] AlRahabi MK (2017). Predictors, prevention, and management of postoperative pain associated with nonsurgical root canal treatment: A systematic review. J. Taibah. Univ. Med. Sci..

[9] Law AS (2015). Predicting severe pain after root canal therapy in the National Dental PBRN. J. Dent. Res..

[10] Ertiaei A (2019). Application of an artificial neural network model for early outcome prediction of gamma knife radiosurgery in patients with trigeminal neuralgia and determining the relative importance of risk factors. Clin. Neurol. Neurosurg..

[11] Stetter BJ (2020). A machine learning and wearable sensor based approach to estimate external knee flexion and adduction moments during various locomotion tasks. Front. Bioeng. Biotechnol..

[12] Choi HI (2019). Artificial intelligent model with neural network machine learning for the diagnosis of orthognathic surgery. J. Craniofac. Surg..

[13] Murata M (2019). Deep-learning classification using convolutional neural network for evaluation of maxillary sinusitis on panoramic radiography. Oral Radiol..

[14] Han HG, Wang LD, Qiao JF (2013). Efficient self-organizing multilayer neural network for nonlinear system modeling. Neural Netw..

[15] Vickram AS (2016). Validation of artificial neural network models for predicting biochemical markers associated with male infertility. Syst. Biol. Reprod. Med..

[16] Oyedotun OK, Olaniyi EO, Khashman A (2016). Disk hernia and spondylolisthesis diagnosis using biomechanical features and neural network. Technol. Health Care.

[17] LaFaro RJ (2015). Neural network prediction of ICU length of stay following cardiac surgery based on pre-incision variables. PLoS One.

[18] Esteva A (2017). Dermatologist-level classification of skin cancer with deep neural networks. Nature.

[19] Johnson A (2019). Use of mobile health apps and wearable technology to assess changes and predict pain during treatment of acute pain in sickle cell disease: Feasibility study. JMIR Mhealth Uhealth.

[20] Wu CC (2019). An artificial intelligence approach to early predict non-ST-elevation myocardial infarction patients with chest pain. Comput. Methods Programs Biomed..

[21] Erickson JD (1973). Statistical tests for the OHI-S and PI: A commentary. J. Dent. Res..

[22] Jonsson Sjögren J, Kvist T, Eliasson A, Pigg M (2019). The frequency and characteristics of pain and discomfort associated with root filled teeth: A practice-based study. Int. Endod. J..

[23] Daly S (2020). A randomised controlled trial to determine patient experience of a magnetostrictive stack scaler as compared to a piezoelectric scaler, in supportive periodontal therapy. J. Dent..

[24] Pak JG, White SN (2011). Pain prevalence and severity before, during, and after root canal treatment: A systematic review. J. Endod..

[25] Manfredi M, Figini L, Gagliani M, Lodi G (2016). Single versus multiple visits for endodontic treatment of permanent teeth. Cochrane Database Syst. Rev..

[26] Hou XM, Su Z, Hou BX (2017). Post endodontic pain following single-visit root canal preparation with rotary vs reciprocating instruments: A meta-analysis of randomized clinical trials. BMC Oral Health.

[27] Farzaneh S, Parirokh M, Nakhaee N, Abbott PV (2018). Effect of two different concentrations of sodium hypochlorite on postoperative pain following single-visit root canal treatment: A triple-blind randomized clinical trial. Int. Endod. J..

[28] Arslan H, Guven Y, Karatas E, Doganay E (2017). Effect of the simultaneous working length control during root canal preparation on postoperative pain. J. Endod..

[29] Zhang X, Lee SY, Luo H, Liu H (2019). A prediction model of sleep disturbances among female nurses by using the BP-ANN. J. Nurs. Manag..

[30] Pivetta T (2013). Development and validation of a general approach to predict and quantify the synergism of anti-cancer drugs using experimental design and artificial neural networks. Talanta.

[31] Hu L (2015). Prediction of liver injury using the BP-ANN model with metabolic parameters in overweight and obese Chinese subjects. Int. J. Clin. Exp. Med..

[32] Ekert T (2019). Deep learning for the radiographic detection of apical lesions. J. Endod..

[33] Demirci F (2016). Artificial neural network approach in laboratory test reporting: Learning algorithms. Am. J. Clin. Pathol..

[34] Bewes J, Low A, Morphett A, Pate FD, Henneberg M (2019). Artificial intelligence for sex determination of skeletal remains: Application of a deep learning artificial neural network to human skulls. J. Forensic Leg Med..

[35] Bas B (2012). Use of artificial neural network in differentiation of subgroups of temporomandibular internal derangements: A preliminary study. J. Oral Maxillofac. Surg..

[36] Schiess M, Urbanczik R, Senn W (2016). Somato-dendritic synaptic plasticity and error-backpropagation in active dendrites. PLoS Comput. Biol..

[37] Lillicrap TP, Cownden D, Tweed DB, Akerman CJ (2016). Random synaptic feedback weights support error backpropagation for deep learning. Nat. Commun..

[38] Lee JH, Kim DH, Jeong SN, Choi SH (2018). Detection and diagnosis of dental caries using a deep learning-based convolutional neural network algorithm. J. Dent..

[39] Shamszadeh S, Shirvani A, Asgary S (2019). Does occlusal reduction reduce post-endodontic pain? A systematic review and meta-analysis. J. Oral Rehabil..

[40] Arias A, de la Macorra JC, Hidalgo JJ, Azabal M (2013). Predictive models of pain following root canal treatment: A prospective clinical study. Int. Endod. J..

[41] Schwendicke F, Golla T, Dreher M, Krois J (2019). Convolutional neural networks for dental image diagnostics: A scoping review. J. Dent..

[42] Muralidharan A, Smith MT (2011). Pain, analgesia and genetics. J. Pharm. Pharmacol..

[43] Schwendicke F, Elhennawy K, Paris S, Friebertshauser P, Krois J (2020). Deep learning for caries lesion detection in near-infrared light transillumination images: A pilot study. J. Dent..

[44] Mohammadi Z, Abbott PV, Shalavi S, Yazdizadeh M (2017). Postoperative pain following treatment of teeth with irreversible pulpitis: A review. N Y State Dent. J..

[45] Ali A (2016). Influence of preoperative pain intensity on postoperative pain after root canal treatment: A prospective clinical study. J. Dent..

[46] Vieyra JP, Enriquez FJ, Acosta FO, Guardado JA (2019). Reduction of postendodontic pain after one-visit root canal treatment using three irrigating regimens with different temperature. Niger. J. Clin. Pract..

[47] Sun C (2018). Pain after root canal treatment with different instruments: A systematic review and meta-analysis. Oral Dis..

[48] Arias A, de la Macorra JC, Azabal M, Hidalgo JJ, Peters OA (2015). Prospective case controlled clinical study of post-endodontic pain after rotary root canal preparation performed by a single operator. J. Dent..

[49] Tait RC (2018). Persistent post-mastectomy pain: Risk factors and current approaches to treatment. J. Pain.

[50] Philpott R (2018). Prevalence, predictive factors and clinical course of persistent pain associated with teeth displaying periapical healing following nonsurgical root canal treatment: A prospective study. Int. Endod. J..

[51] Konermann A (2017). In vivo determination of tooth mobility after fixed orthodontic appliance therapy with a novel intraoral measurement device. Clin. Oral Invest..

[52] Meirelles L (2020). Quantitative tooth mobility evaluation based on intraoral scanner measurements. J. Periodontol..

